# Liposomal Formulation of Bioactive Substances from *Mangifera indica* Peels for Potential Cosmetic Applications

**DOI:** 10.3390/ijms27135934

**Published:** 2026-07-01

**Authors:** Nika Kučuk, Mateja Primožič, Željko Knez, Maja Leitgeb

**Affiliations:** 1 Faculty of Chemistry and Chemical Engineering, University of Maribor, Smetanova ulica 17, 2000 Maribor, Slovenia; nika.kucuk1@gmail.com (N.K.); mateja.primozic@um.si (M.P.); zeljko.knez@um.si (Ž.K.); 2 Faculty of Medicine, University of Maribor, Taborska ulica 8, 2000 Maribor, Slovenia

**Keywords:** liposomes, synthesis, zeta potential, mango peels, encapsulation, *in vitro* release, antibacterial activity

## Abstract

Several sensitive bioactive substances are potent antioxidants that protect the skin from free radicals but are often rapidly degraded, limiting their effectiveness. Encapsulating these substances in liposomes improves their bioavailability and solubility and protects them from harmful environmental factors. The influence of liposomes as advanced lipid nanocarriers is increasing enormously due to their remarkable properties and protection of bioactive substances. For this reason, mango (*Mangifera indica* L.) peel extract (MPE), previously characterized and rich in various natural substances, including ellagic acid, gallic acid, and catechin, has been encapsulated in liposomes. The investigation focused on the impact of different liposome synthesis process parameters on their size, size distribution, stability, and encapsulation efficiency, and on *in vitro* release as a potential advanced MPE delivery system with suitable characteristics. An important study on the influence of the organic solvent used in liposome synthesis on the above properties is described. The thin lipid film hydration method using 5-mm glass beads and ethanol as an organic solvent was the most favorable method for synthesizing a stable and monodisperse lipid–MPE delivery system. MPE was successfully encapsulated in liposomes with the highest encapsulation efficiency of 53.7%. The sustained release of MPE from the liposomes was achieved, and the antibacterial properties of MPE, incorporated into the liposomes, were retained. For the first time, MPE has been encapsulated in liposomes, and with the remarkable results obtained, the extract represents a formulation with high added value that can be used in various fields, especially for the enrichment of different products such as cosmetic creams and lotion.

## 1. Introduction

Liposomes are self-assembled spherical lipid nanocarriers consisting of one or more concentric phospholipid bilayers and a hydrophilic core [1,2]. They are biocompatible, biodegradable, non-toxic, non-immunogenic, and non-pathogenic. Due to their unique and beneficial properties, they belong to the most important and promising delivery systems [3,4,5]. Liposomes can successfully entrap various unstable therapeutic substances, which are important for human health [6], allowing their stabilization and thus preserving their functionality. They effectively ensure the protection of the encapsulated active substances from enzymatic degradation, chemical and immunological inactivation, and rapid plasma clearance. In addition, undesirable properties of the substances, such as low solubility and poor membrane permeability, are improved. This consequently leads to their stability and the improvement with prolongation of their half-life [7,8]. Furthermore, at the target site, significant enhancement of the therapeutic effect (in the biodistribution) can be observed [9,10]. Therefore, liposomes have emerged as promising delivery systems for various therapeutic agents with different biomedical applications.

There are various well-developed methods for the successful synthesis of liposomes, divided into mechanical methods (thin lipid film hydration, the freeze–thaw, sonication, the French press cell, membrane extrusion, and micro-emulsification) and solvent dispersion methods (reverse-phase evaporation, ethanol injection, ether injection (solvent vaporization), and double emulsification) [11,12,13]. Depending on the preparation technique and post-processing steps, liposomes may be obtained as multilamellar vesicles (MLVs) or as unilamellar vesicles, including small unilamellar vesicles (SUVs), large unilamellar vesicles (LUVs), and giant unilamellar vesicles (GUVs). Conventional thin-film hydration typically produces MLVs, whereas additional processing methods such as sonication, extrusion, or high-pressure homogenization are frequently applied to reduce particle size and generate predominantly unilamellar vesicles. Therefore, both the preparation method and post-processing conditions influence the final size and lamellarity of liposomal formulations. However, particle size alone does not determine lamellarity, and dedicated structural characterization is required to distinguish between multilamellar and unilamellar liposomes [14,15].

The choice of a suitable method for the preparation of liposomes is influenced by various factors, such as the physicochemical properties, concentration, and toxicity of the encapsulated therapeutic agents, the encapsulation efficiency, the sustained release at the target site, the choice of organic solvent used to disperse the lipids, the stability, size, and polydispersity of the liposomes obtained, and the synthesis cost [16]. However, the thin lipid film hydration method is the most commonly used technique [17,18].

**Figure 1:**
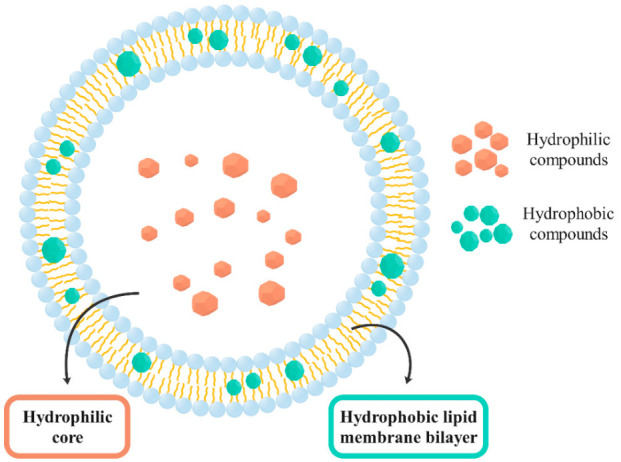
General liposome structure and possible incorporation of hydrophilic and hydrophobic compounds.

Liposomes have a significant advantage over other nanocarriers because they can successfully encapsulate and release molecules with different solubilities; both hydrophilic and hydrophobic components. Hydrophilic compounds can be incorporated into the hydrophilic core of the liposomal vesicle, while hydrophobic molecules can be incorporated into the lipid bilayer membrane [19,20,21], as schematically presented in Figure 1.

Furthermore, liposomes are among the best-known cosmetic carrier systems for cosmetic formulations, i.e., for cosmetic products with bioactive substances with medical benefits [22]. A crucial positive feature is the biocompatibility with human tissue due to the structural similarity between the lipid bilayer and the natural biological membrane [23], as liposomes can be synthesized from various naturally occurring substances. Therefore, liposomes are not perceived as foreign by the body. The lipid membrane reduces the risk of acute or chronic toxicity as it consists of physiological lipids, an excellent advantage for using liposomes in cosmetics [24]. The main components of the liposomal lipid bilayer are phospholipids such as phosphatidylcholine and sterols, especially cholesterol, which are generally recognized as safe (GRAS) [25]. Phosphatidylcholine is also used in many hair and skin care products for its conditioning and emollient properties. Soy and vegetable phospholipids are particularly interesting for dermatology and cosmetics due to their content of highly esterified essential fatty acids [22].

Liposomes for cosmetic applications play an essential role as carriers of bioactive cosmetic substances and also as active agents. Unloaded liposomes, i.e., liposomes without encapsulated bioactive substances, can act on skin lipids, proteins, and carbohydrates and thus help the damaged skin to return to normal and the stratum corneum to properly fulfill its defense function [26]. A clinical report showed that liposomes help to smooth the skin and reduce wrinkles [22].

Sensitive natural biologically active substances can be degraded too quickly, resulting in limited bioavailability. Encapsulation in various vehicles plays a crucial role, as it successfully prevents evaporation and protects the encapsulated material from unfavorable environmental conditions. Thus, the degradation of the plant material and its bioactive substances is reduced. Encapsulation can take place in various advanced delivery systems [27,28,29,30], e.g., in liposomes, micelles, dendrimers, (bio)polymeric nanoparticles, and inorganic nanoparticles, which enable the delivery of the incorporated substances to the target sites and prevent possible toxic effects on healthy cells [31,32].

As carriers of bioactive substances in cosmetic formulations, liposomes have a multifunctional role, i.e., in addition to their own positive effect, they also provide better skin penetration, solubility, and stability, reduce toxicity, and enable the delivery of encapsulated bioactive substances to the target site and provide a sustained release profile [26]. Due to their ability to alter the fluidity of the cell membrane and fuse with the cells, liposomes can efficiently deliver encapsulated bioactive substances to the target site [33].

Various bioactive substances act as powerful antioxidants and protect the skin from the harmful effects of free radicals [34]. These are usually stable and bioactive in plants but can be degraded after extraction due to their sensitivity to light and heat [35]. Encapsulation plays an important role in increasing their bioavailability and solubility as it protects the encapsulated material from adverse environmental influences [27,29]. In recent years, liposomes have been extensively studied as delivery systems and used for the encapsulation of various bioactive substances in order to achieve a stronger effect on the target cells. They have already proven to be promising carriers of various bioactive substances, such as curcumin [36], quercetin [37], resveratrol [38], apigenin [39], caffeic acid [40], gallic acid [41], and rutin [42]. In our previous study, we demonstrated that liposomes can be used for biomedical applications to achieve sustained release of gallic acid, with an encapsulation efficiency of 98% [41]. In the context of vitamin encapsulation, both, hydrophilic and hydrophobic vitamins, liposomes are among the most commonly used nanocarriers [43].

In addition, the successful encapsulation of essential oils (Barije [44], clove [45], rosemary [46], thyme [47], Greek sage [48], cinnamon [49], oregano [50]) in liposomes was also investigated and confirmed that they are protected from degradation by UV light and that the biological activity, in particular the antioxidant and antimicrobial activity, is retained. Liposomes have also been shown to be efficient carriers of plant extracts such as rosehip [51], garlic [52], white tea [53], black carrot [54], moringa [55], betel leaves [56], and bamboo leaves [57].

On the other hand, constant population growth, economic growth, lifestyle changes, and dietary habits are contributing to a dramatic increase in food produced worldwide. As a result, significant amounts of agro-industrial waste are generated, including inedible parts of fruits, which pose a heavy burden on the environment if not correctly disposed of or not further used [58,59,60]. One of these fruits is mango, one of the most widely consumed tropical fruits in the world. As a result, large quantities of mango by-products, such as seeds and peels, are produced. Mango peels make up 15 to 20% of the total weight of the fruit [61,62]. On the other hand, mango peels are not merely an agricultural by-product but a highly valuable source of various bioactive substances, including vitamins, minerals, fiber, carotenoids, and phenolic compounds. Several studies have shown that mango peel contains significantly higher concentrations of phenolic compounds and antioxidants than the edible pulp [63,64,65,66]. These compounds exhibit strong antioxidant, antimicrobial, anticancer, anti-inflammatory properties, and skin-protective properties, making mango peels an attractive raw material for cosmetic and biomedical applications [61]. However, many of these phytochemicals are sensitive to environmental conditions such as light, oxygen, temperature, and pH, which can reduce their stability and bioavailability during storage and application.

Encapsulation within liposomal carrier systems offers a promising strategy to overcome these limitations by protecting sensitive phytochemicals from degradation, improving their stability and controlled release, and enhancing their interaction with biological membranes. Therefore, mango peel extract represents a particularly suitable candidate for liposomal delivery systems.

Furthermore, the use of mango peel in advanced liposomal formulations should not be viewed merely as waste disposal. Instead, it represents a value-added valorization approach aligned with circular economy principles and pollution reduction, in which low-value agro-industrial residues are transformed into high-value functional ingredients. The combination of waste-derived bioactive compounds with nanocarrier technology enables the development of innovative formulations with potential applications in cosmetics and personal care products, contributing simultaneously to sustainability, resource efficiency, and product innovation.

Liposomes with encapsulated extracts from fruit waste, which without further use represent a significant burden on the environment, are also a possible application in new formulations for the pharmaceutical and cosmetics industries. The successful encapsulation of grape seeds [67], pomegranate peels [68], and citrus peels [69] has already been reported in the literature. However, despite the high content of bioactive phenolic compounds and the growing interest in mango peel valorization, research on the encapsulation of mango (*Mangifera indica* L.) peel extract (MPE) in liposomal systems is still scarce. This highlights an important research gap, particularly in view of the potential synergistic benefits of integrating waste-derived bioactive compounds with advanced nanocarrier technologies for cosmetic applications.

Liposomes with incorporated MPE represent a promising delivery system that can be used in various fields, especially in the cosmetics industry, to enrich cosmetic products. Namely, MPE is an excellent source of polyphenolic compounds, in particular, ellagic acid, gallic acid, and catechin [63]. The cosmetic potential of MPE is closely related to its phytochemical composition. Ellagic acid has been reported to inhibit melanogenesis and reduce hyperpigmentation through modulation of tyrosinase activity, making it a promising ingredient for skin-brightening formulations. Gallic acid exhibits strong antioxidant and anti-inflammatory properties and may contribute to protecting skin cells against oxidative stress induced by ultraviolet radiation. Catechin and related flavonoids have also been associated with antioxidant, photoprotective, anti-aging, and anti-inflammatory effects through their ability to scavenge reactive oxygen species and modulate inflammatory pathways. Collectively, these bioactive compounds may contribute to maintaining skin homeostasis, reducing premature skin aging, and improving the overall performance of cosmetic formulations. However, their susceptibility to environmental degradation may limit their effectiveness, highlighting the importance of suitable delivery systems such as liposomes [70,71,72,73,74]. Therefore, our study focused for the first time on optimizing the synthesis of liposomes with suitable properties as advanced nanocarriers for bioactive substances from MPE. The antibiotic ciprofloxacin (CIP) was used as a reference drug.

The influence of different parameters related to the synthesis method on the characteristics of liposomes in terms of stability, particle size, and size distribution, as well as on the encapsulation efficiency and the *in vitro* release of previously characterized MPE, for which phytochemical composition and biological activity had been demonstrated in our previous study [63], was investigated. The thermal stability of MPE encapsulated in liposomes and possible chemical interactions between MPE and the lipids of the phospholipid bilayer, as well as morphological characteristics of functionalized liposomes, were determined. The time-dependent inhibitory effect on the growth of bacterial species, Gram-negative *Escherichia coli* and Gram-positive *Staphylococcus aureus*, was also investigated. The antibacterial activity of functionalized liposomes with MPE is important to improve cosmetic products’ stability, efficacy, and safety. This enables the development of advanced formulations that protect the skin, extend product shelf life, and meet consumer demand for effective and gentle skin care solutions.

## 2. Results and Discussion

In our previous study [63], the phytochemical and biological characterization of MPE obtained from dried mango peels using UAE with EtOH was comprehensively investigated. The extract demonstrated good antibacterial activity against both Gram-negative (*E. coli*, *Pseudomonas aeruginosa*) and Gram-positive bacterial species (*Bacillus cereus*, *S. aureus*), as well as significant antioxidant activity. Furthermore, liquid chromatography–mass spectrometry (LC-MS/MS) analysis confirmed the presence of several bioactive phenolic compounds, including caffeic acid, chlorogenic acid, gallic acid, catechin, and hesperidin/neohesperidin. MPE also proved to be valuable source of protein and certain enzymes in a highly active form, such as α-amylase, cellulase, glucoamylase, laccase, lipase, polyphenol oxidase and superoxide dismutase.

Based on the demonstrated phytochemical composition and biological activity of MPE in our previous study [63], this extract was selected as a promising candidate for encapsulation into liposomal carrier systems. Therefore, the present study aimed to optimize the production of liposomes as potential carrier systems to protect sensitive bioactive substances and to preserve the bioactivity of MPE for use in various biomedical applications.

### 2.1. Liposome Preparation Through Thin Lipid Film Hydration Method

#### 2.1.1. Influence of the Synthesis Method on the Characteristics of Liposomes

First, the optimization was focused on the liposome synthesis method to obtain stable liposomes with uniform size distribution. Namely, the properties of the liposomes produced are strongly influenced by the synthesis method used [75]. The liposomes were prepared using soybean phosphatidylcholine and cholesterol at a ratio of 3:1 (*w*/*w*) by the thin lipid film hydration method, as described in Section 3.4. The use of soybean phosphatidylcholine, which contains predominantly unsaturated fatty acids, contributes to membrane fluidity and may influence the physicochemical characteristics of the resulting liposomal formulations. This method is based on the dissolution of the lipids in an organic solvent, evaporation of the organic solvent under reduced pressure, formation of a thin lipid film, hydration of the film with an aqueous media, successive agitation or stirring, reducing the size of the liposomes, post-formation process (purification), and characterization of the nanoformulated product [75,76]. Ethanol (EtOH) was used as an organic solvent with 2 h of shaking at 200 rpm, and the addition of glass beads. Glass beads of different sizes (3 or 5 mm) were added to investigate their influence on the size and stability of the synthesized liposomes. For comparison, a conventional method with sonication was used, as described in Section 3.4. The obtained liposomal formulations regarding particle size, polydispersity index (PDI), and zeta potential were evaluated. The results obtained are summarized in Table 1.

Liposomes were successfully synthesized regardless of the synthesis method used. Mean particle size, PDI, and zeta potential were determined using the dynamic light scattering (DLS) technique. The size of the synthesized liposomes is one of the most important and critical criteria. It affects the stability, biodistribution, cellular uptake, entrapment efficiency, and release profile of the encapsulated substance [77]. Based on their size, liposomes can be divided into SUVs (size range 20–100 nm), LUVs (size 100–1000 nm), and GUVs (size > 1000 nm) [14,15]. As seen from Table 1, obtained liposomes exhibited diameters in the range of 400–600 nm, which falls within the size range commonly associated with large vesicles (100–1000 nm). However, because lamellarity was not directly assessed in this study, no conclusions can be drawn whether the vesicles were unilamellar or multilamellar based on the particle size measurements alone.

The smallest liposome size was obtained when shaking with 3 mm glass beads was used, while the largest diameter was obtained with the sonication method. Teong et al. [78] also report liposome sizes of over 400 nm when using the sonication procedure. Another important parameter is the PDI, which determines the particle size distribution. The PDI value indicates the heterogeneity of the liposome formulation in terms of particle size [79]. The PDI value can vary between 0, indicating a completely uniform or monodisperse liposomal formulation, and 1, indicating a highly polydisperse liposomal formulation. The lowest PDI value and, thus, the least polydisperse liposomal formulation was obtained by shaking with 5 mm glass beads. The density of the 3 mm glass beads is lower, so the required shear force cannot be provided to achieve a more uniform particle size distribution [76]. According to the PDI values determined, which are closer to 1, the synthesized liposomes by shaking with 3 mm glass beads and sonication have a greater tendency to aggregate, which is very undesirable for further applications.

In addition, the zeta potential, which is based on the surface charge of the particles (mV), is a crucial parameter for determining the stability of liposomes. Zeta potential values in the range between 0 and 10 mV and between 0 and −10 mV indicate a very unstable formulation Values in the range between 10 and 20 mV and between −10 and −20 mV indicate a relatively stable formulation Values in the range between 20 and 30 mV and between −20 and −30 mV indicate a moderately stable formulation, and zeta potential values above ±30 mV indicate a very stable liposomal formulation where aggregation of the particles is successfully prevented [80,81]. All three liposomal formulations proved to be stable, as the zeta potential was below −30 mV. Although the liposomal formulation obtained by sonication was the most stable with a zeta potential of −85.10 mV, the particles were large and very heterogeneous, which is undesirable from the biomedical applications point of view. The addition of glass beads enables the synthesis of a monodispersed liposomal system. Also, the glass beads can be reused, and no further procedures are required to reduce the size of the liposomes and for purification, which is a great advantage from a time and economic point of view [76].

The size of liposomes for different biomedical applications is preferably between 50 and 500 nm [75]. Only the liposomes synthesized by shaking with 3 mm glass beads met this criterion, but their size distribution was insufficient. Specifically, the PDI obtained under these initial conditions indicated a relatively broad particle size distribution and therefore did not represent the optimal formulation. Therefore, the shaking method with 5 mm glass beads with the determined lower PDI value was further optimized by extending the shaking time and thus increasing the shear force. This optimization strategy was undertaken to improve liposome homogeneity and reduce polydispersity. The effect of shaking time on the size of the synthesized liposomes as potential drug carriers (liposome size < 200 nm [3]) was investigated. The shaking time was extended to 5 and 24 h. The results obtained are shown in Table 2.

In the case of glass beads with a larger diameter, sufficient shear forces cannot be guaranteed with a shorter shaking time due to the larger interstitial space. If the shaking time is extended, i.e., longer than the shear forces are applied, liposomes with smaller average sizes can be synthesized [76], as was achieved in our study (Table 2). After 24 h of shaking, the average diameter of the liposomes was 142.53 nm, which is a favorable size for their use as delivery systems (<200 nm [3]). After shaking for 5 h and 24 h, a homogeneous and monodisperse formulation was obtained based on the PDI values since the criterion determines that the system is considered homogeneous if the PDI value is 0.3 or less [82]. Although by extending the shaking time the stability of the liposomes did not change significantly, the determined zeta potential values indicated a stable liposome system.

From the results of the DLS analysis, it can be concluded that shaking for 24 h was the most favorable method for producing a stable and monodisperse liposomal formulation with the appropriate mean particle diameter. Similar observations regarding the more relevant characteristics of liposomes under prolonged exposure to shear forces were made in the study by Wang et al. [76].

#### 2.1.2. Influence of the Organic Solvent on the Characteristics of Liposomes

**Figure 2:**
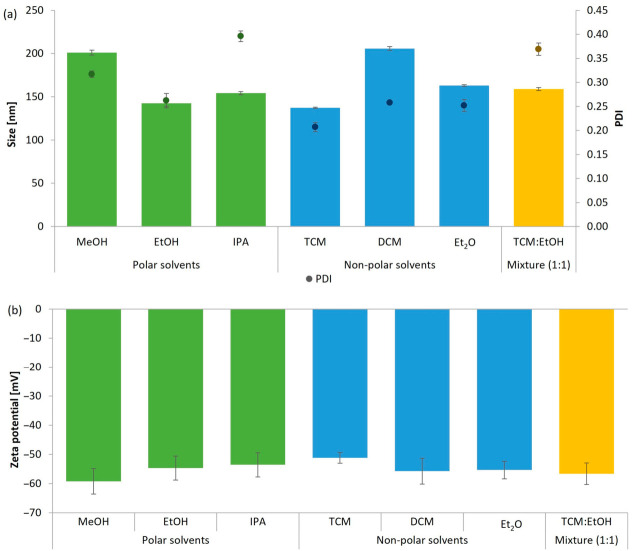
Characteristics of liposomal formulations in terms of (**a**) size and PDI, and (**b**) zeta potential using different organic solvents in the synthesis procedure.

The choice of organic solvents in the dissolution of lipids for liposome synthesis is a crucial factor affecting the efficiency, stability, size, and overall quality of liposomes. Studying the effects of different solvents can lead to optimized liposome formulations tailored to specific applications in drug delivery, cosmetics, or other fields [83]. Therefore, the influence of the organic solvent used to dissolve the lipids (phosphatidylcholine and cholesterol) on the properties of the synthesized liposomes was further investigated using the optimized synthesis method (thin lipid film hydration method with shaking for 24 h in the presence of 5 mm glass beads). It was found that the properties of the individual organic solvent used in the lipid dissolution phase can have a decisive influence on the physiochemical characteristics of the synthesized liposomes. Differences in the characteristics can arise due to the different polarities of the organic solvents [84]. Therefore, three polar solvents, methanol (MeOH), ethanol (EtOH), and isopropyl alcohol (IPA), and three non-polar solvents, chloroform (trichloromethane—TCM), dichloromethane (DCM) and diethyl ether (Et_2_O) were used. The influence of a mixture of polar (EtOH) and non-polar solvent (TCM) in a 1:1 ratio was also investigated, which, according to the DLS analysis, led to the best characteristics of the liposomes. The physicochemical properties of the prepared liposomes, determined in terms of particle size, polydispersity index (PDI), and zeta potential, are presented in Figure 2. To provide a systematic overview of the optimization process, the figure illustrates the influence of the investigated preparation parameters on these characteristics. This graphical presentation enables direct comparison of the different synthesis conditions and facilitates visualization of the observed trends, which served as the basis for selecting the optimized liposome preparation protocol.

When polar solvents were used, the sizes of the synthesized liposomes followed the order of the used solvents MeOH > IPA > EtOH. The PDI values also followed the same sequence. The best characteristics, i.e., suitable particle size and homogeneous particle population, were achieved using EtOH (142.53 nm; PDI = 0.262), which also fulfills the criteria for possible use in biotechnological applications for drug delivery systems [3].

Depending on the non-polar solvent used, the size of the synthesized liposomes and the PDI values obtained follow the sequence of the used solvents DCM > Et_2_O > TCM (Figure 2a). However, all these liposomal formulations were homogeneous or monodisperse, as all PDI values were below 0.3. Overall, the most desirable characteristics were obtained when TCM was used, i.e., the average size of the synthesized liposomes was the smallest (137.40 nm), as was the PDI value (0.207).

Since liposomes synthesized with EtOH (liposomes (EtOH)) among the polar solvents and TCM (liposomes (TCM)) among the non-polar solvents showed the most favorable characteristics, the influence of the mixture EtOH:TCM (1:1) on the characteristics of the synthesized liposomes (liposomes (EtOH:TCM)) was investigated. However, this resulted in the formation of liposomes with a larger average diameter and a non-uniform size distribution (PDI > 0.3).

MeOH is the most polar of the solvents tested (0.762), followed by EtOH (0.654) and IPA (0.546). Webb et al. [85] found that the reduction in alcohol polarity (MeOH > EtOH > IPA) in the microfluidic production of liposomes led to an increase in particle size. However, in our study and in the study of Obeid et al. [84], no similar effect was observed with respect to the polarity of the solvent, as no direct correlation was found between the polarity of the solvent and the size of the synthesized particles. The same observation applied to non-polar solvents, as the particle size did not depend on the decreasing polarity (TCM > DCM > Et_2_O). It was found that the size of the synthesized liposomes can be influenced by the type of organic solvent but not by the polarity of the solvent.

Furthermore, the stability of the liposomes was not influenced by the choice of organic solvent (Figure 2b). It can be confirmed that all synthesized liposomal formulations were stable regardless of the organic solvent used, as the zeta potential values determined were quite comparable and ranged between −51.17 and −59.23 mV. EtOH and TCM were selected as organic solvents for further analysis because they exhibited the best physiochemical properties in terms of the smallest liposome size and most homogeneous liposome formulation among the polar and non-polar solvents, respectively.

### 2.2. Influence of Organic Solvent on the Encapsulation Efficiency of Bioactive Substances in Liposomes and In Vitro Release

Since the choice of organic solvent influences the physical properties of liposomes, it is important to consider the use of a specific organic solvent for the potential use of loaded liposomes in cosmetic applications. Therefore, MPE or the antibiotic CIP as a reference drug were encapsulated in liposomes, and the influence of the organic solvent on the physical properties of liposomes, encapsulation efficiency, and *in vitro* release of bioactive substances from liposomes were studied. EtOH and TCM were chosen as organic solvents among the polar and non-polar solvents, respectively, as their use showed the best physicochemical characteristics of liposomes in terms of smallest particle size, stability and most homogeneous liposome formulation. These physical characteristics, especially the size of the liposomes, have a major influence on the dermal delivery of the encapsulated compounds into the skin [86]. Additionally, despite the slightly poorer characteristics of unloaded liposomes obtained with a mixture of EtOH and TCM (1:1) compared to those obtained with single solvents, the influence of this mixture on the encapsulation of bioactive substances was also investigated. For the synthesis of liposomes, the previously optimized method of thin lipid film hydration with shaking for 24 h in the presence of 5 mm glass beads was used. The results of the DLS analysis are shown in Table 3; the results for empty liposomes from Section 2.1.2 are presented for comparison.

The localization of encapsulated compounds within liposomes depends largely on their physicochemical properties. MPE is a complex mixture composed predominantly of polar phytochemicals extracted with ethanol, including phenolic acids and flavonoids, although compounds with intermediate lipophilicity may also be present. Consequently, many of its constituents are expected to preferentially partition into the hydrophilic core of the liposomes, whereas less polar molecules may associate with the phospholipid bilayer. CIP is an amphoteric antibiotic with appreciable aqueous solubility under the formulation conditions employed in this study and is therefore expected to be predominantly localized in the hydrophilic core. However, the precise localization of MPE constituents and CIP within the liposomal structure was not directly investigated in the present work and would require dedicated analytical techniques for confirmation.

Encapsulation of MPE solution (2.5 mg/mL) in liposomes resulted in a more than twofold increase in mean liposome size compared to unloaded liposomes, independent of the used organic solvent. Since the MPE solution contained hydrophilic molecules, it was entrapped in the aqueous inner core of the liposomes, which was the reason for the increase in particle size. Another reason was the fluidization effect of the potentially present hydrophobic molecules, which leads to disturbances in the phospholipid bilayer due to the possible insertion of phytochemical substances [87]. An increase in particle size distribution was also observed. Our results are consistent with other studies, in which an increase in liposome size was also observed when various extracts were encapsulated, such as extracts of *Centella asiatica* leaves [88], *Lycium barbarum* leaves [87], *Teucrium montanum* L. [89] and *Crithmum maritimum* [90]. The most favorable properties of synthesized liposomes in terms of the smallest particle size and the lowest PDI value were obtained when EtOH was used.

On the other hand, these properties were achieved with TCM in the CIP-loaded liposomes. In addition, only a slight increase in particle size was observed when encapsulating CIP solution (0.1 mg/mL) compared to unloaded liposomes. The explanation for this lies in the fact that antibiotics are small molecules [91] compared to the wide variety of large bioactive molecules present in MPE. The CIP-loaded liposomal formulations were considered monodisperse systems as the PDI values were below 0.3.

The concentrations of MPE (2.5 mg/mL) and CIP (0.1 mg/mL) used in this study were selected for different experimental purposes and were not intended to provide directly comparable loading conditions. CIP served primarily as a reference compound, whereas MPE was investigated as the target natural extract for cosmetic applications. The substantially higher concentration and chemically complex composition of MPE may have contributed to the observed increase in particle size through interactions with the lipid bilayer and the encapsulation process. Therefore, differences in the physicochemical properties of the MPE- and CIP-loaded liposomes should be interpreted in the context of both their distinct compositions and their different loading concentrations.

All zeta potential values determined were comparable and below −30 mV, indicating the production of stable liposomal formulations. A more negative zeta potential leads to a stronger electrostatic repulsion between the particles, which successfully reduces the possibility of particle aggregation. In addition, the high surface charge increases the interaction between liposomes and cells, which ensures better delivery of the encapsulated bioactive substances [6,88].

**Figure 3:**
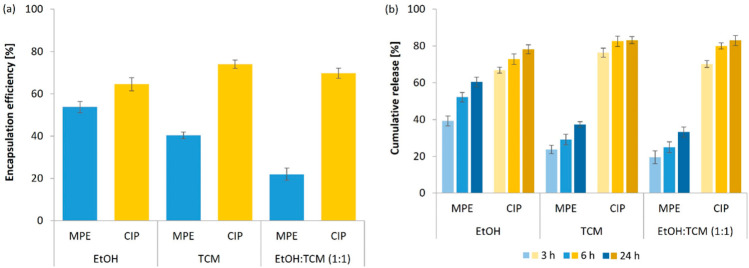
(**a**) Encapsulation efficiency of MPE and CIP in liposomes using different organic solvents (EtOH, TCM, and a mixture of EtOH:TCM (1:1)) for liposome synthesis and (**b**) the percentage release of MPE and CIP from liposomes at different time intervals.

The characteristics of liposomes have a direct influence on the stability and bioavailability of encapsulated bioactive substances from extracts [89]. In the optimization of extract-loaded liposomal formulations, encapsulation efficiency is one of the most important physicochemical factors, as the bioavailability of the extracts and their bioactive substances can be ensured by a high encapsulation efficiency [92,93]. The encapsulation efficiency (Figure 3a) of MPE and CIP in liposomes and *in vitro* release study (Figure 3b) were further evaluated using EtOH, TCM, or a mixture of EtOH:TCM (1:1).

It is evident that there was also a significant impact of the used organic solvent on the encapsulation efficiency of MPE. A polar and non-polar solvent mixture proved to be the least suitable choice. Most of the MPE was entrapped into liposomes when EtOH was used as a solvent (53.7%). Other authors also reported the successful encapsulation of various extracts in liposomes using the thin lipid film hydration method and EtOH as the organic solvent, but without using glass beads, e.g., from betel leaves (59–80%) [56], *Malus hupehensis* (76–80%) [94], *Centella asiatica* leaves (40–68%) [88] and mountain germander (46–52%) [89]. However, to the best of our knowledge, no studies have been available on the use of glass beads for uniform liposome synthesis with encapsulated extracts. In other studies, much emphasis is generally placed on using different synthesis methods such as homogenization or extrusion as well as sonication, which, however, proved to be unsuitable in our study. In contrast, the difference in encapsulation efficiency of CIP in liposomes (64.6–74.0%) was not as pronounced as that of MPE when different solvents were used. However, the highest value (74.0%) was achieved using TCM as a solvent. The small size of the CIP molecules makes it easier to encapsulate CIP in liposomes compared to MPE. Consequently, higher encapsulation efficiencies were obtained for CIP. However, using EtOH as a solvent resulted in suitably MPE-loaded liposomes appropriate for cosmetic applications.

As a further step, the *in vitro* release of MPE and CIP encapsulated in liposomes was studied in phosphate-buffered saline (PBS) at constant shaking (100 rpm) at 37 °C. The percentage of CIP and MPE released after 3, 6, and 24 h using the dialysis technique is shown in Figure 3b. The release of the encapsulated active ingredients from the liposomal carriers is a consequence of rupture, destabilization due to local defects in the bilayer membrane [95,96]. Encapsulated active ingredients should be released in an appropriate amount, close to the target site, and at a certain time, which is directly related to the increase in bioavailability and the elimination of possible adverse effects of the active ingredients [77].

When exposed to PBS at a temperature of 37 °C, the structure of the liposomes changed, allowing the encapsulated bioactive substances to escape successfully. The release profile of CIP showed an initial rapid release (3 h) independent of the organic solvent used in contrast to the release of MPE. Thereafter, the release of CIP stabilized and did not increase by more than 15% up to 24 h. In contrast to CIP, MPE was released more slowly and exhibited a prolonged release profile. The most favorable release profile was determined for the liposomes synthesized with EtOH when comparing the organic solvents, EtOH, TCM, and their mixture in a 1:1 ratio. MPE was released more slowly from the liposomes synthesized with TCM and the mixture, and even after 24 h the percentage of MPE released did not exceed 40%, while more than 60% was released from the liposomes prepared with EtOH.

Overall, the choice of organic solvent in liposome production can also have a direct impact on the safety and efficacy of the synthesized liposomal formulation as well as on consumer acceptance of the final cosmetic product, making it a critical factor in the development process. The most favorable characteristics of liposomes in terms of particle size, size distribution, stability, encapsulation efficiency, and release profile were obtained in the encapsulation of MPE when EtOH was used in the liposome synthesis for the dissolution process of lipids. This is a very favorable result because EtOH is non-toxic and harmless to humans and the environment. It is also considered GRAS and can be used safely in biomedical, food, and cosmetic applications.

In this context, the effects of MPE concentration on liposome characteristics were further investigated and are presented in the Section 2.3. In contrast, these characteristics were obtained using TCM in the preparation of CIP-loaded liposomes.

### 2.3. Influence of MPE Concentration on the Characteristics of Liposomes

**Figure 4:**
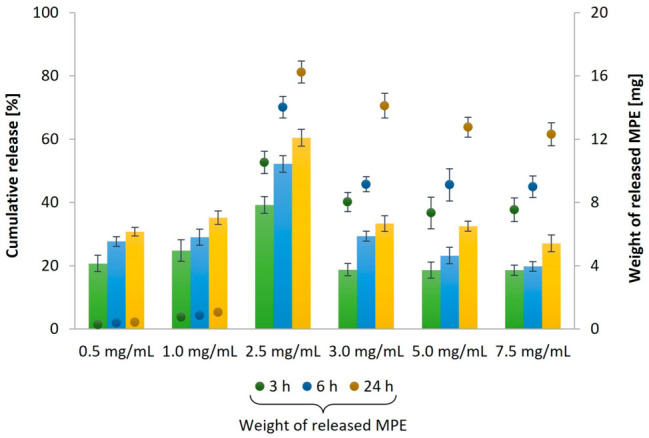
Release profile of MPE solutions at different concentrations from liposomes.

The influence of the MPE concentration (0.5–7.5 mg/mL) on the properties of the liposomes in terms of particle size, size distribution, stability, encapsulation efficiency, and loading capacity (Table 4), as well as the release profile at 3, 6 and 24 h (Figure 4), was investigated.

The results indicate that the size of the liposomes as expected increased with increasing MPE concentration, coinciding with other studies on the encapsulation of different extracts [56,97]. The higher the amount of extract, the more it can be incorporated into the interior, into the aqueous core, and also into the lipid layer or on the outside, which leads to an enlargement of the mean size of liposomes. On the other hand, uneven encapsulation can directly affect the particle size distribution. However, the PDI values did not differ significantly, with the most homogeneous particle distribution achieved when encapsulating MPE into liposomes at a concentration of 2.5 mg/mL, which corresponds to the range of PDI values (0.2–0.3) for carriers prepared from biological materials [90].

At this concentration, the highest encapsulation efficiency was also achieved. A decrease in encapsulation efficiency was observed with a further increase in the MPE concentration. The same observation was made by Guldiken et al. [98]. However, according to the dry weight of the synthesized liposomes, as the concentration of the MPE increased, a larger amount of the MPE was entrapped in the liposomes. Furthermore, the concentration of the encapsulated MPE had no significant influence on the stability of the liposomes. All liposomal formulations were stable, with strong electronegative membrane surface charge.

Regarding the release of MPE of different concentrations from liposomes in PBS at 37 °C and constant stirring at 100 rpm (Figure 4), a prolonged release took place, being the most uniform for the MPE solution with a concentration of 2.5 mg/mL. Generally, liposomes containing a lower amount of the extract showed a slower release rate [56], as was confirmed also in our study. However, the release was also less efficient at the higher tested concentrations (3.0–7.5 mg/mL) as a smaller amount of the MPE was released from the liposomes. For example, at an encapsulated concentration of 7.5 mg/mL, more than 30% less MPE was released from the liposomes than at 2.5 mg/mL. Indeed, the amount of MPE loaded into the liposomes affected the release rate, as a slower release rate was observed in liposome formulations with lower encapsulation efficiency, similar to a study by Tagrida et al. [56]. The higher the amount of encapsulated MPE, the lower the percentage of released MPE. The explanation lies in the packaging density of the phospholipids in the liposome bilayers, which is the reason for the lower permeability of the encapsulated bioactive substances [77].

Based on the study on the effect of MPE concentration on the liposome’s characteristics, it was found that the requirements for the potential use of loaded liposomes in various biomedical applications are best met with a concentration of 2.5 mg/mL. It has been estimated that suitable particle size for topical application in the cosmetic industry has been achieved (<300 nm) [99], which has beneficial properties in enhancing skin penetration and delivery of bioactive substances into the skin layers, improving the efficiency of the cosmetic product due to the increased bioavailability of the encapsulated bioactive substances and ensuring a uniform and consistent application. A favorable zeta potential, which measures the surface charge of the particles, and appropriate size distribution were also achieved, indicating a monodisperse formulation (PDI < 0.3). This means that the particles are less likely to aggregate over time, contributing to consistent performance and long shelf life. Penetration and interaction with the skin can also be better predicted and controlled [79]. Additionally, the highest percentage of encapsulation efficiency was achieved, ensuring effective protection of bioactive substances and the most desirable release profile, allowing controlled and sustained release, thereby enhancing the performance of the cosmetic product. Therefore, liposomes with MPE at a 2.5 mg/mL concentration were used for further analysis.

### 2.4. Morphological Characteristics of Liposomes

**Figure 5:**
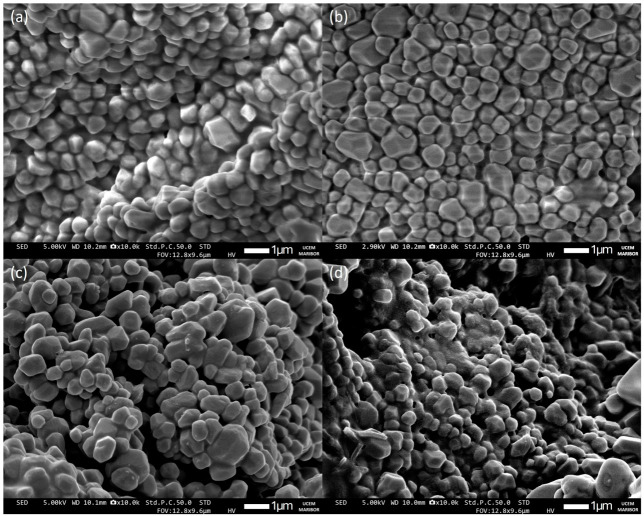
SEM images of freeze-dried unloaded liposomes prepared using (**a**) EtOH or (**b**) TCM and liposomes with encapsulated (**c**) MPE using EtOH or (**d**) CIP prepared using TCM at 10,000× magnification.

Liposomes are generally used for various applications suspended in an aqueous solution, usually in a buffer such as PBS. However, they can degrade rapidly in aqueous solutions, which limits their shelf life and thus their usefulness for biomedical applications. Therefore, various drying techniques, such as spray drying or freeze drying, are often used to extend the shelf life of liposomes [100]. Using scanning electron microscopy (SEM) analysis, the morphological characteristics of freeze-dried unloaded liposomes (EtOH or TCM) and liposomes with encapsulated MPE or CIP were investigated, as shown in Figure 5.

During freeze-drying (Figure 5), the structure of the liposomes was slightly deformed, as they no longer had the spherical shape typical of liposomes [101]. In addition, their surface was slightly wrinkled, which is a consequence of the rapid shrinkage of the sample due to the cooling process during freeze-drying [88]. Similarly, Ramli et al. [102] observed the deformed and wrinkled surface of unloaded liposomes and liposomes with encapsulated propolis extract.

The average diameter of liposomes after freeze-drying increased compared to the particle size in fresh liposome suspension (results of DLS analysis). This is most likely due to the aggregation of the particles during the freeze-drying process. The average size of the unloaded liposomes (EtOH) increased from 142.53 ± 3.41 nm (before freeze-drying) to 545.43 ± 128.69 nm (after freeze-drying), and that of the empty liposomes (TCM) from 137.40 ± 0.70 nm to 677.70 ± 169.38 nm. An increase in liposome size was also observed for functionalized liposomes, from 288.33 ± 3.96 nm to 734.60 ± 264.88 nm for MPE-loaded liposomes and from 128.10 ± 1.95 nm to 627.71 ± 222.04 nm for CIP-loaded liposomes. Large values of the standard deviation confirm an inhomogeneous size distribution. El-Nesr et al. [103] also reported the fusion or aggregation of liposomes during the freeze-drying process. They further confirmed that the addition of cryoprotectants during freeze-drying can successfully prevent liposome aggregation. In this way, the same size distribution of liposomes is maintained. However, we can confirm the excellent stability of the freeze-dried liposomes, as no degradation was observed in the obtained SEM images, indicating their stability even in the solid form.

### 2.5. Thermal Stability of Liposomes

**Figure 6:**
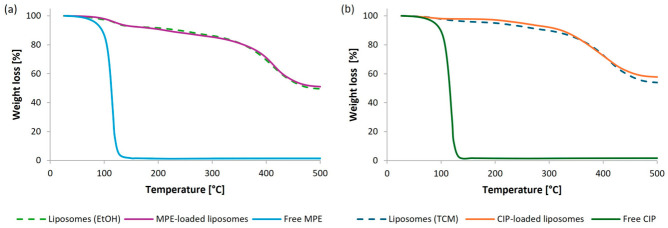
TGA thermograms of (**a**) empty liposomes (EtOH), MPE-loaded liposomes, and free MPE and (**b**) empty liposomes (TCM), CIP-loaded liposomes, and free CIP.

Since bioactive substances are generally heat-sensitive and unstable, encapsulation in carriers such as liposomes is crucial. In addition, heating has a significant impact on the carriers and leads to alterations such as dehydration, oxidation, combustion, and decomposition [104]. Therefore, thermal stability of the synthesized empty and loaded liposomes as well as the free MPE and CIP, was determined by thermogravimetric analysis/differential scanning calorimetry (TGA/DSC) analysis. Thermal degradation of the samples is shown in Figure 6 as TGA thermograms, which show the weight loss in a temperature range of 25–500 °C.

The TGA thermogram of free bioactive substances presents a drastic weight loss of 80.8% in the temperature range from 63 to 159 °C of the free MPE (Figure 6a) and 69.3% from 60 to 165 °C of the free CIP (Figure 6b). Both resulted in the final percentage weight loss of over 98% at 150 °C. Unloaded liposomes synthesized with EtOH and MPE-loaded liposomes showed a two-phase weight reduction. The first phase of weight loss between 35 and 214 °C resulted in a weight reduction of 8.7% for the empty liposomes (EtOH) and 8.6% for the MPE-loaded liposomes in a temperature range from 43 to 191 °C. This could be due to the loss of moisture. During the final weight loss, a weight reduction of 41.5% (215–500 °C) and 39.5% (193–498 °C) was observed for unloaded liposomes (EtOH) and MPE-loaded liposomes, respectively. This weight loss could be due to changes in the molecular structure causing a phase transition [105]. No significant differences were found in the weight loss profile between unloaded liposomes (EtOH) and MPE-loaded liposomes. Also, no significant differences in weight loss between empty liposomes obtained with TCM and CIP-loaded liposomes were observed. There was a gradual loss of sample weight throughout the entire temperature range, namely 44.6% for unloaded liposomes (TCM) and 39.8% for CIP-loaded liposomes.

**Figure 7:**
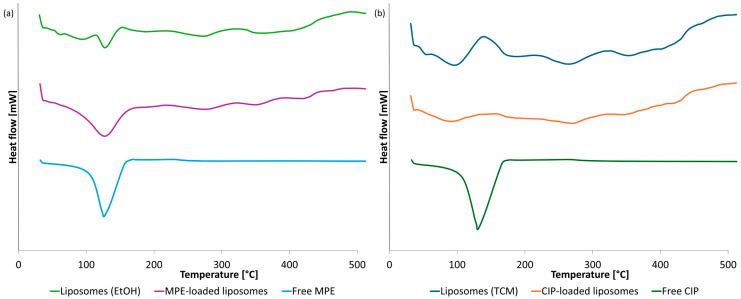
DSC thermograms of (**a**) empty liposomes (EtOH), MPE-loaded liposomes, and free MPE and (**b**) unloaded liposomes (TCM), CIP-loaded liposomes, and free CIP.

Furthermore, the phase behavior of the samples was investigated using DSC analysis in the temperature range from 25 to 500 °C. The DSC thermograms of free MPE and CIP, as well as empty and loaded liposomes, are shown in Figure 7.

From the DSC thermogram of free MPE and free CIP, a sharp endothermic peak can be noticed at 120 and 123 °C, respectively.

Unloaded liposomes (EtOH) showed endothermic peaks at 88, 122, 179, 268, and 342 °C. Other studies also report a sharp endothermic peak and several thermal events [51,106]. However, the DSC thermogram of MPE-loaded liposomes showed that the peak position shifted to slightly higher temperatures. The first and the sharpest peak was observed at 123 °C. Less pronounced endothermic peaks at 189, 273, 346, and 410 °C indicate further thermal events associated with the decomposition of the sample.

In addition, the use of different organic solvents also influenced the thermal stability of the liposomes, as liposomes (EtOH) exhibited endothermic peaks at higher temperatures than liposomes (TCM).

An endothermic peak was observed at 93 and 84 °C of unloaded liposomes (TCM) and CIP-loaded liposomes, respectively, due to the hot movement of the polar head group of the phospholipids [107]. This was followed by several thermal events with less pronounced endothermic peaks at 169, 253, and 342 °C for unloaded liposomes (TCM) and at 169, 263 and 337 °C for CIP-loaded liposomes, which is associated with a phase transition [107] and further degradation of the lipid matrix [51,108].

Thermal analysis can provide detailed information on the stability of the loaded liposomes during the synthesis process and storage [51]. The results indicated that the thermal stability of MPE was improved with the encapsulation of MPE into liposomes, which is in accordance with other studies on the incorporation of extracts of *Cordyceps sinensis* CS1197 [105]. In addition, previous studies found a shift in the peak position in the DSC curve can be observed for liposomes with encapsulated extracts [107,109,110]. The bioactive substances, including flavonoids, act as spacers within the lipid bilayer, leading to destabilization and a decrease in the temperature at which the transition from the gel phase to the liquid–crystal phase occurs [51].

Although the present study demonstrated favorable thermal stability and physicochemical characteristics of the developed liposomes, additional factors relevant to long-term storage should also be considered. In particular, phospholipids containing unsaturated fatty acid chains are susceptible to oxidative degradation upon prolonged exposure to oxygen and light, which may compromise membrane integrity and the stability of the encapsulated compounds. The present study did not evaluate oxidative or photostability during extended storage, and this represents a limitation of the work. Future investigations should therefore assess storage stability under various environmental conditions and explore strategies such as light-protective packaging, freeze-drying, storage under an inert atmosphere, or the incorporation of suitable antioxidants to maximize the shelf life of the formulation for cosmetic applications.

### 2.6. FTIR Analysis

**Figure 8:**
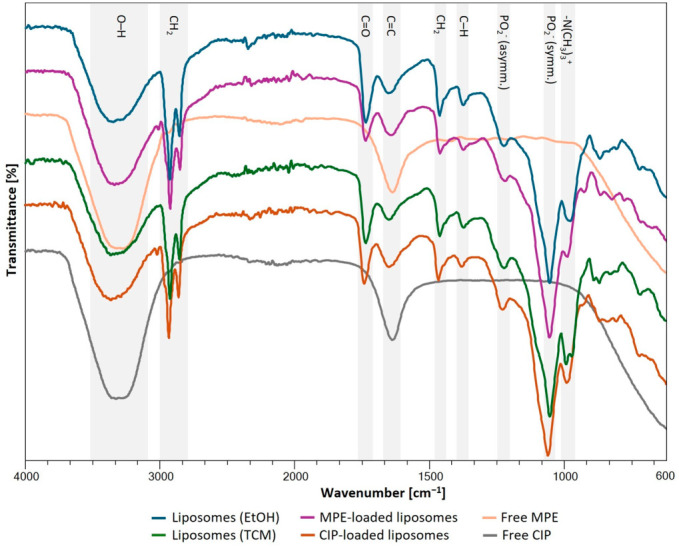
FTIR spectra of empty liposomes (EtOH and TCM), MPE- and CIP-loaded liposomes, and free MPE and CIP.

Fourier transform infrared spectroscopy (FTIR) analysis in the range from 400 to 4000 cm^−1^ was performed to study the occurrence of possible molecular interactions between the encapsulated MPE or CIP and the lipids of the phospholipid bilayer, which are reflected in the FTIR spectra (Figure 8). The interaction between the components is reflected in a change in the wave number or in the intensity of the absorption peaks of lipids and encapsulated bioactive substances. The concentration of molecules or ions (functional groups) in the sample determines the intensity of the infrared spectra [77].

Unloaded liposomes obtained with either EtOH or TCM showed characteristic peaks of liposomes. A broad peak 3500–3200 cm^−1^ corresponded to hydroxyl (O-H) stretching vibrations and intermolecular hydrogen bonding. Asymmetric and symmetric CH_2_ stretching in the methyl and methylene groups of alkyl chains were observed at 2926 and 2853 cm^−1^, respectively. The peak at 1736 cm^−1^ can be assigned to the ester groups (C=O), which is a typical band for phosphatidylcholine [111]. Further, the peak at 1647 cm^−1^ (both) is associated with the alkene carbon-carbon double bond C=C. Peaks at 1466 cm^−1^ and 1464 cm^−1^, and peaks at 1377 cm^−1^ and 1375 cm^−1^ corresponded to the scissoring bending vibrations of the CH_2_ groups and umbrella deformation of C-H vibrations (methyl groups of alkyl chains), respectively. Also, the characteristic vibrations of the phosphate groups were visible, namely, PO_2_^−^ asymmetric and PO_2_^−^ symmetric stretching modes at 1219 cm^−1^ and 1223 cm^−1^, respectively, and both at 1053 cm^−1^. In addition, the antisymmetric –N(CH_3_)_3_^+^ stretching vibration was also observed at 976 cm^−1^ and 970 cm^−1^ [112]. These results are in accordance with FTIR spectra obtained in other reported studies [77,111,112,113,114,115].

The FTIR spectrum of pure MPE showed characteristic peaks at 3271 cm^−1^ corresponding to the O-H stretching vibration, at 2947 cm^−1^ and 2859 cm^−1^ due to asymmetric and symmetric CH_2_ stretching vibrations, and the peak at 1636 cm^−1^ was due to the stretching vibrations of the C=C groups. The peaks in the 1600–1200 cm^−1^ range were consistent with the presence of polyphenols in MPE [116]. The FTIR spectrum of CIP also indicated peaks at 3314 cm^−1^ (O-H stretching vibrations), at 2930 cm^−1^ and 2853 cm^−1^ (CH_2_ asymmetric and symmetric stretching vibrations), and at 1636 cm^−1^ (stretching vibrations of the C=C groups), similar as in a study by Khan et al. [117].

The characteristic peaks of MPE- and CIP-loaded liposomes in the FTIR spectrum appeared at 3500–3200 cm^−1^ (O-H stretching vibrations), both at 2924 cm^−1^ (symmetric CH_2_ stretching), both at 2853 cm^−1^ (antisymmetric CH_2_ stretching), both at 1740 cm^−1^ (C=O ester groups), 1638 and 1636 cm^−1^ (C=C bond), 1458 and 1466 cm^−1^, (scissoring bending vibrations of CH_2_ groups), 1375 and 1377 cm^−1^ (deformation of C-H vibrations), 1217 and 1221 cm^−1^ (PO_2_^−^ antisymmetric mode), both 1055 cm^−1^ (PO_2_^−^ symmetric mode), and 991 and 984 cm^−1^ (asymmetric –N(CH_3_)_3_^+^ stretching vibration).

Some differences were found in the absorption peaks of the loaded liposomes compared to the unloaded liposomes, which was probably because the functional groups of the encapsulated bioactive substances overlapped with the bands of the unloaded liposomes [111]. However, no strong interactions between bioactive substances and liposomes were detected, indicating confirmation of encapsulation and good compatibility between the lipid matrix and the bioactive substance. Other researchers have also drawn similar conclusions regarding negligible chemical interactions between encapsulated extracts and liposomes [77,111], suggesting that liposomes represent an advanced and promising nanocarrier system for plant extracts.

### 2.7. In Vitro Release Kinetics of Encapsulated Bioactive Substances from Liposomes

Liposomes have significant potential as carriers for bioactive substances and monitoring their release is important to ensure effective delivery. The *in vitro* release study of bioactive substances from liposomes is primarily designed to evaluate their release over time under controlled conditions. This study provides information on the behavior of the liposomal formulation, including the release rate and extent of release of the loaded bioactive substances, their stability and their interaction with the environment [118]. For the cosmetics industry, understanding the release behavior of bioactive substances encapsulated in liposomes is critical to ensure a controlled and sustained release that improves performance and extends the stability and shelf life of cosmetic products [119,120].

**Figure 9:**
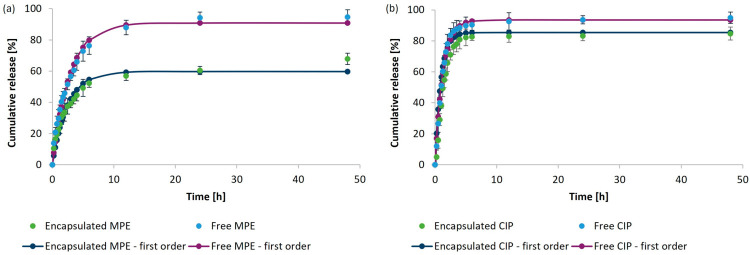
Cumulative release profiles of MPE (**a**) and CIP (**b**), together with the corresponding fitted theoretical release curves for liposome-encapsulated compounds. The diffusion of free MPE and free CIP through the dialysis membrane into the dialysate, determined using the dialysis technique, is also shown for comparison.

Therefore, the *in vitro* release study was performed over 48 h in PBS at pH 7.4, 37 °C, and 100 rpm [76,121]. For comparison, a study was also conducted in which MPE or CIP in free form was passed through the dialysis membrane into the dialysate in a time-dependent manner. The results are presented in Figure 9a and Figure 9b for MPE and CIP, respectively.

The release profiles of MPE- and CIP-loaded liposomes were evaluated to characterize the behavior of the respective formulations under identical experimental conditions. MPE and CIP were incorporated at different concentrations, and CIP served primarily as a reference formulation. Therefore, the observed release patterns provide qualitative insight into the sustained-release characteristics of liposomal encapsulation and into the influence of the physicochemical properties of the encapsulated materials.

The release of encapsulated MPE was slightly slower and more gradual than the passage of non-encapsulated MPE through the dialysis membrane. The MPE was gradually released from the liposomes for up to 6 h, with 57.0% of the encapsulated MPE released, which corresponds to 14.03 mg of released MPE in the dialysate. Thereafter, the release rate was slower. The transition of free MPE into the dialysate was almost completely stopped after 24 h (94.2%, 47.11 mg), as the percentage of MPE released increased by less than 0.5% up to 48 h. The release also stabilized after 6 h for the encapsulated MPE. However, a consistent release profile was still detectable up to 48 h, when 67.8% of the MPE was released, corresponding to 18.22 mg of MPE released.

The sustained release behavior observed for MPE is consistent with the structural characteristics of liposomal delivery systems. Hydrophilic constituents are expected to be predominantly retained within the aqueous core of the vesicles, whereas amphiphilic and hydrophobic molecules may partition into or associate with the phospholipid bilayer. Consequently, their release occurs through diffusion across the lipid membrane and gradual membrane reorganization rather than by immediate liberation into the surrounding medium. In addition, the incorporation of cholesterol into the bilayer decreases membrane permeability and enhances structural integrity, thereby moderating the release rate of encapsulated compounds. Beyond controlling release kinetics, the lipid bilayer acts as a physical barrier that reduces the exposure of sensitive phytochemicals to environmental factors such as oxygen, light, and elevated temperatures, thereby improving their stability and helping to preserve their biological activity during storage and application.

We can confirm the successful encapsulation of MPE in liposomes and the more favorable and desirable release profile of MPE when incorporated into the liposomal carrier system, which is very promising for various biomedical applications. Particularly with regard to incorporation into various cosmetic products, liposomes would not only improve the stability and protection of the bioactive substances in MPE against degradation, but could also enable deeper penetration into the skin [26], which would further enhance the positive effects of the bioactive substances. The controlled release profile can provide a long-lasting effect on the skin, reducing the need for frequent application and increasing user satisfaction. In addition, liposomes can minimize skin irritation and enable targeted delivery, which improves the efficacy of encapsulated bioactive substances in the treatment of certain skin conditions. In particular, they can maintain their antioxidant properties and thus improve skin health [26,122,123]. This offers a natural, biocompatible, and innovative solution for modern skin care formulations.

Since MPE is rich in bioactive substances, especially ellagic acid, gallic acid, and catechin [63], which are potent antioxidants, MPE-loaded liposomes could effectively combat oxidative stress, reduce the appearance of wrinkles, and protect the skin from environmental damage. While liposomes enabled a controlled release of bioactive compounds from MPE in our study, the release can be sustained over a more extended period. This is very beneficial for cosmetics, especially for brightening the skin, preventing hyperpigmentation, fighting wrinkles and aging, and reducing inflammation, which are one of the important effects of ellagic acid, gallic acid, and catechin on the skin [70,71,72,73,74]. In addition, the results of our study are consistent with other investigations indicating a prolonged release of various extracts from the liposomal formulation [56,87,124].

On the other hand, a completely different release profile was observed for encapsulated CIP from liposomes and the profile of passage through the pores of the dialysis membrane of free CIP. In the first 3 h, a burst release of encapsulated CIP and the passage of free CIP through the dialysis membrane into the dialysate was observed. Thus, 76.4% of the encapsulated CIP passed through liposomes, and 86.3% of the free CIP through the dialysis membrane into the dialysate. Since CIP is a small molecule, it rapidly escaped from inside the liposomes when the bilayer membrane of the liposomes was ruptured, indicating a release profile similar to the passage of free CIP through the dialysis membrane. Thereafter, the release of CIP followed a constant release profile. After 48 h of exposure to PBS at 37 °C, the release of encapsulated CIP was 84.7% efficient, corresponding to a release of 1.09 mg of encapsulated CIP. The percentage of free CIP in the dialysate after 48 h was 94.9%, corresponding to a total mass of 1.73 mg CIP in the dialysate.

In order to better understand the release mechanism and behavior of bioactive substances from liposomes in comparison to substances in free form and to predict *in vivo* performance, four different kinetic models were fitted to the experimental data. The zero-order, first-order, Higuchi, and Korsmeyer–Peppas modes were used, which are most frequently used to describe the release behavior of active substances from liposomes [125]. Based on the correlation coefficients (*R*^2^), the kinetic model that best fitted the experimental data obtained in the period from 0 to 48 h was selected. The kinetic parameters and *R*^2^ determined are presented in Table 5.

It can be stated that the best correlation of the experimental results for all samples corresponds to the first-order kinetic model, as the *R*^2^ value is closest to 1 for all samples studied. The first-order model indicates the concentration-dependent release of bioactive substances from liposomes and the passage of bioactive substances in free form into the dialysate through the pores of the dialysis bag. The release rate of bioactive substances slows down over time as their concentration decreases.

**Figure 10:**
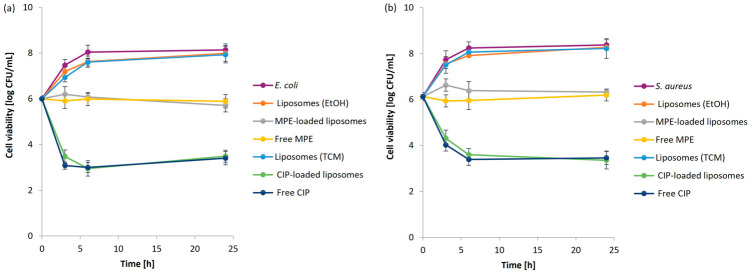
Growth curves of (**a**) *E. coli* and (**b**) *S. aureus* in the presence of unloaded liposomes (EtOH or TCM), MPE- or CIP-loaded liposomes, and free MPE or CIP.

Additionally, Figure 10 shows the experimental results adapted to the theoretically predicted release of bioactive compounds from liposomes or the passage of bioactive compounds in free form into the dialysate through the pores of the dialysis bag according to the first-order release mechanism. In general, the release of various therapeutic agents from liposomes is expected to follow first-order kinetics [126], which is consistent with our results. The observed constant time release kinetics is advantageous in the administration of therapeutic agents for various biomedical and other applications [127]. The n-parameter (diffusion exponent) in the Korsmeyer–Peppas model provides additional information for predicting the release mechanism and the behavior of encapsulated substances [124]. If *n* < 0.45 applies, the release corresponds to Fickian diffusion, if 0.45 < *n* < 0.85 to non-Fickian diffusion or anomalous diffusion, if *n* = 0.85 the release mechanism belongs to Case II transport, and if *n* > 0.85 to Super Case II transport for spherical systems, including liposomes [128,129]. The experimentally determined *n*-values for all samples are below 0.45, which corresponds to the Fickian diffusion mechanism in which the transport of molecules is associated with a concentration gradient [130]. This is in line with release profiles from other studies, where the release of polyphenols from extracts also followed a Fickian diffusion [87,124].

### 2.8. Antibacterial Properties of Loaded Liposomes

In our previous study [63], the outstanding inhibitory properties of MPE on the growth of Gram-negative (*E. coli* and *Pseudomonas aeruginosa*) and Gram-positive bacterial species (*Bacillus cereus* and *S. aureus*) was already confirmed, indicating a successful synergistic effect of the various polyphenolic compounds detected in MPE.

Due to the possible rapid degradation of the extracts and sensitive bioactive substances, it is necessary to encapsulate them in order to preserve their bioactivity, including their antimicrobial activity. Liposomes are, therefore, suitable carriers for the encapsulation of bioactive substances. They enable a controlled and prolonged release, which can lead to sufficient antimicrobial activity of the encapsulated compounds over a certain period of time [5,131].

MPE-loaded liposomes and free MPE were subjected to an antimicrobial study using the plate count method. The differences in the inhibitory effect of the samples on the growth of *E. coli* and *S. aureus* were studied throughout 24 h at 37 °C. CIP-loaded liposomes and free CIP were used as a positive reference. The results of a kinetic study of the bacterial growth curves are presented in Figure 10a and Figure 10b for *E. coli* and *S. aureus*, respectively, expressed as cell viability (log CFU/mL).

Table 6 summarizes the percentages of bacterial reduction upon exposure of *E. coli* and *S. aureus* to free and encapsulated bioactive substances after 3, 6, and 24 h of incubation, calculated from the results presented in Figure 10.

The unloaded liposomes (EtOH) and (TCM) exhibited a similar trend against bacterial growth as the reference bacterial suspension. This indicates that the lipid composition of the liposome bilayer membrane did not influence bacterial growth. On the other hand, both pathogens were successfully inhibited when exposed to free and encapsulated MPE and CIP, respectively. Liposomes loaded with MPE and CIP showed a similar killing curve as the free forms of MPE and CIP. However, in the presence of the compounds encapsulated in liposomes, a more uniform inhibition occurred at the beginning of the study, indicating a gradual bursting of the liposomes and the associated release of bioactive substances with antimicrobial activity.

The results are consistent with the results of the *in vitro* release study. After three hours, 39.2% of the encapsulated MPE was released from the liposomes, while 56.7% of the free MPE passed through the pores of the dialysis bag (Figure 9a), whereby the percentage reduction for both *E. coli* and *S. aureus* (Table 6) with the encapsulated MPE was correspondingly lower compared to the free MPE. The longer the bacteria were exposed to the samples, the more MPE was released, and the more effective inhibition occurred. After 6 h when more than half of the encapsulated MPE was released (52.2%) and 76.4% of the free MPE was passed through the dialysis membrane into the dialysate, a high percentage of bacterial reduction was achieved (>97%). When the incubation time of the microbial populations was further extended (24 h), no increase in the percentage of bacterial reduction was observed (>98), regardless of the test sample.

The preservation of antibacterial activity after encapsulation indicates that the liposome preparation process did not adversely affect the bioactive phytochemicals responsible for the antimicrobial properties of MPE. In addition, the phospholipid bilayer may protect sensitive phenolic compounds from degradation, thereby helping to preserve their biological activity.

The same applies to CIP, which was already released to more than 80% after 4 h (Figure 9b), after which the release stabilized. The results are consistent with the study of antimicrobial activity, as complete inhibition of the growth of the two tested bacteria was achieved after only three hours.

Other authors have also reported the effective antimicrobial activity of liposomes loaded with extracts and pure bioactive substances using different methods. Some studies estimated that loaded liposomes even showed a higher inhibition rate at lower minimum inhibitory concentrations (MIC) compared to free substances [49,132,133]. Liposomes loaded with antibiotics such as moxifloxacin [134], gentamicin [135], vancomycin [136], and azithromycin [137] also proved to be successful antimicrobial nanocarriers.

However, due to the inappropriate and excessive use of antibiotics, various pathogenic bacterial species that cause serious diseases are becoming increasingly resistant [138,139]. Therefore, natural extracts and bioactive substances with potential antibacterial properties are receiving more and more attention [140]. Liposomes with encapsulated antibacterial extracts, such as MPE, can improve their bioavailability and enable more effective treatment of skin diseases [141,142,143]. They, therefore, represent an effective alternative and can potentially reduce the risk of bacteria developing resistance. As shown in our study, MPE-loaded liposomes can provide long-lasting protection against bacteria due to the sustained release of bioactive substances from MPE and are, therefore, a promising strategy for the production of highly effective natural cosmetic products [144,145]. In addition, liposomes with encapsulated extracts can also reduce the risk of irritation or side effects, which makes them particularly interesting for sensitive skin [146], especially with regard to MPE, as it is rich in various bioactive compounds.

Although the present study did not include *in vitro* or *in vivo* skin efficacy experiments, the cosmetic relevance of the developed formulation is supported by the established biological activities of the major phenolic constituents previously identified in MPE, including ellagic acid, gallic acid, and catechin. These compounds have been widely reported to possess antioxidant, anti-inflammatory, photoprotective, and skin-brightening properties. The successful encapsulation of MPE in liposomes, together with the demonstrated sustained release profile and preservation of antibacterial activity, suggests its potential applicability in cosmetic formulations. Nevertheless, further studies involving skin cell models, skin permeation experiments, and clinical evaluations are necessary to confirm the cosmetic efficacy of the developed formulation.

The preservation of antibacterial activity following encapsulation suggests that the liposomal formulation maintains the functional integrity of the bioactive constituents responsible for microbial growth inhibition. However, this activity should not be attributed to a single compound but rather to the combined effects of multiple phytochemicals present in MPE, including phenolic acids and flavonoids identified in our previous compositional analysis [63]. Thus, liposomal encapsulation appears to preserve the overall antibacterial potential of the extract while simultaneously providing controlled release of the incorporated constituents. Nevertheless, the present study does not distinguish the individual contributions of specific compounds to the observed antibacterial activity. Future studies aimed at identifying the relative contributions of individual constituents and evaluating their release-dependent antimicrobial effects would provide further insight into the mechanisms underlying this biological activity.

## 3. Materials and Methods

### 3.1. Chemicals and Reagents

The following chemicals: ethanol (EtOH, ≥99.9%), hydrochloric acid (HCl, 37.0%), meat extract, meat peptone, potassium dihydrogen phosphate (KH_2_PO_4_), potassium chloride (KCl), sodium dihydrogen phosphate (Na_2_HPO_4_) and sodium chloride (NaCl) were purchased from Merck, Darmstadt, Germany. Tryptic soy broth was obtained from Fluka, Buchs, Switzerland. D-(+)-glucose anhydrous was purchased from Kemika, Zagreb, Croatia. Agar, chloroform (TCM, ≥99.0%), cholesterol (≥99.0%), dichloromethane (DCM, ≥99.5%), diethyl ether (Et_2_O, ≥99.8%), methanol (MeOH, ≥99.9%), phosphatidylcholine (≥99.0%), peptone from soybean, 2-propanol (IPA, ≥99.8%), sodium hydroxide (NaOH, ≥95.0%) and yeast extract were purchased from Sigma-Aldrich, St. Louis, MO, USA. Ciprofloxacin (CIP, 400 mg/200 mL) was obtained from the University Medical Centre Maribor, Maribor, Slovenia.

### 3.2. Microorganisms

Selected bacterial species *E. coli* (DSM 498) and *S. aureus* (DSM 346) were purchased from DSMZ-German Collection of Microorganisms and Cell Cultures GmbH from Berlin, Germany.

### 3.3. Ultrasound-Assisted Extraction of Mango Peels

Ultrasound-assisted extraction (UAE) was used for the preparation of MPE from air-dried mango peels (Keitt type), which was described in detail in our previous study [63].

### 3.4. Liposome Synthesis

Liposomes were prepared using a thin lipid film hydration method. Soybean phosphatidylcholine and cholesterol at a ratio of 3:1 (*w*/*w*) were dissolved in an organic solvent. When using polar solvents, constant stirring and heating up to 50 °C were required to dissolve the lipids. The organic solvents were completely removed under reduced pressure using a rotary evaporator. The resulting thin lipid film on the flask wall was hydrated with an MPE or CIP solution as well as with PBS in the case of empty liposomes. Following a previous study, glass beads of different sizes (3 or 5 mm) were added [76], and the flasks were shaken at 20 °C and 200 rpm for different time periods. In the sonication method, the hydrated lipids were exposed to an ultrasonic bath (VEVOR, Rancho Cucamonga, CA, USA) operating at a frequency of 40 kHz at room temperature for 30 min [78,147].

### 3.5. Characterization of Liposomes by Dynamic Light Scattering Analysis

Liposomes were characterized by DLS analysis using a Zetasizer Nano ZS instrument (Malvern Instruments Co., Malvern, UK) at room temperature. The average particle size and particle size distribution, expressed as PDI, were determined, as well as the zeta potential to determine stability by measuring the surface charge of the liposomes.

### 3.6. Encapsulation Efficiency Determination

The encapsulation efficiency of MPE or CIP in liposomes was determined by the direct method, as described by Risaliti et al. [48]. It was calculated as the ratio between the weight of MPE or CIP in the liposomes and the weight of MPE or CIP in the initial solution.

### 3.7. Scanning Electron Microscopy

SEM analysis was performed using a scanning electron microscope (FE SEM, JEOL JSM-IT800 Schottky, JEOL Ltd., Tokyo, Japan) to examine the morphology of the freeze-dried empty liposomes and liposomes with encapsulated bioactive substances. Before analysis, the liposomes were coated with a conducting carbon layer.

### 3.8. Thermogravimetric Analysis/Differential Scanning Calorimetry

Liposomes were subjected to thermal stability analysis by TGA/DSC performed simultaneously on a TGA/DSC instrument (TGA/DSC1, Mettler Toledo AG (MTANA), Zurich, Switzerland) at a nitrogen flow and temperature range of 25 to 500 °C and a temperature rate of 10 °C/min.

### 3.9. Fourier Transform Infrared Spectroscopy

FTIR analysis determined possible chemical interactions between liposomes and encapsulated MPE and CIP. The freeze-dried liposomes were pressed between two ART diamond crystals. FTIR spectra were acquired in the 4000–600 cm^−1^ range and recorded using the FTIR spectrophotometer (Perkin Elmer 1600, PerkinElmer Inc., Waltham, MA, USA).

### 3.10. In Vitro Release Kinetics of Encapsulated Bioactive Substances

The *in vitro* release of MPE and CIP from liposomes was evaluated in PBS (0.01 M, pH 7.4) at 37 °C using the dialysis technique under continuous shaking at 100 rpm. Prior to the release study, non-encapsulated MPE or CIP was removed from the liposomal suspensions, as described in Section 2.6. At predetermined time intervals, aliquots of the external release medium were collected and analyzed spectrophotometrically using a UV–VIS spectrometer (Varian Cary Probe 50, Agilent Technologies, Santa Clara, CA, USA). After each sampling, the withdrawn volume was immediately replaced with an equal volume of fresh PBS to maintain sink conditions throughout the experiment. The cumulative release (%) of MPE or CIP was calculated as the ratio between the amount of compound released from the liposomes at a given time point and the initial amount encapsulated within the liposomes. For comparison, the diffusion of free MPE and free CIP through the pores of the dialysis membrane was also evaluated under the same experimental conditions.

The kinetics of the MPE or CIP release from liposomes was evaluated using different mathematical models (zero-order, first-order, Higuchi model, and Korsmeyer–Peppas model) [124,148] using software-supported statistical analysis (OriginPro^®^ (version 10.1.0.178, OriginLab Corporation, Northampton, MA, USA)). The zero-order model indicates the percentage of substance released per unit of time), the first-order model illustrates the logarithmic relationship between the remaining percentage of substance and time, the Higuchi model expresses the percentage of substance released as a function of the square root of time, and the Korsmeyer–Peppas model represents the logarithmic relationship between the percentage of substance released and time [30]. The equations used for the kinetic models are as follows (Equations (1)–(4)):

*Q* = *k*_0_ × *t*, (1)

*Q* = *a* × (1 − *e*^−*k*_1_×*t*^), (2)

*Q* = *k_H_* × *t*^0.5^, (3)

*Q* = *k_KP_* × *t^n^*, (4)

where is:

*Q*—release rate at time t (%).

*k*_0_—zero-order release constant (%/min).

*k*_1_—first-order release constant (min^−1^).

*k_H_*—Higuchi model release constant (%/min^1/2^).

*k_KP_*—Korsmeyer–Peppas model release constant (min^−n^).

*t*—time (min).

*a*—asymptotic constant (%).

*n*—relaxation exponent (/).

### 3.11. Determination of Antibacterial Activity

A plate count method was used to evaluate the inhibitory properties of the loaded liposomal formulation compared to the free bioactive substances (MPE or CIP as a reference drug). The antibacterial activity was evaluated against *E. coli*, a representative of Gram-negative bacteria, and *S. aureus*, a representative of Gram-positive bacteria. The bacterial suspensions (10^6^ CFU/mL) were incubated for 24 h at 37 °C in the presence of the samples. At different time intervals (3, 6, and 24 h), the number of viable bacterial colonies using tenfold serial dilutions and the spread plate technique was determined. The size of the bacterial population and CFU/mL were calculated after 24-h incubation at 37 °C. The bacterial reduction percentage was calculated based on the ratio of the difference between the number of colony-forming units per mL of the control (CFU_control_/mL) and the tested sample (CFU_test_/mL) and the number of units of the control sample (CFU_control_/mL).

All experiments were performed in triplicate, and the results are presented as mean values. The study was designed to evaluate formulation behavior and identify overall trends among the investigated systems. Although formal statistical hypothesis testing was not performed, the replicate measurements demonstrated good reproducibility and consistent trends, supporting the reliability and robustness of the reported observations.

## 4. Conclusions

Our study represents an optimization of the process parameters of the synthesis method used to develop liposomes with suitable characteristics as advanced nanocarriers for MPE. As a reference, the antibiotic CIP was encapsulated in liposomes for comparison. The thin lipid film hydration method with glass beads with a diameter of 5 mm and shaking for 24 h proved to be the most suitable method of obtaining a stable delivery system and an appropriate particle size with homogeneous size distribution. Not only the size of the glass beads and the shaking time but also the choice of organic solvent and the concentration of the MPE solution play a decisive role in the characteristics of the liposomes in terms of mean particle size, size distribution, stability, encapsulation efficiency, and *in vitro* release of MPE from liposomes.

Among the six different organic solvents used, EtOH, a non-toxic and harmless organic solvent, yielded the most favorable characteristics of liposomes meeting the criteria for potential drug delivery applications, with a zeta potential < −30 mV, mean particle size < 300 nm, and PDI < 0.3. It was shown that MPE could be successfully encapsulated in liposomes with an encapsulation efficiency of 53.7%, as well as CIP with an encapsulation efficiency of 74.0%. Sustained *in vitro* release over 48 h was achieved, following the first-order kinetic model. In addition, the liposomes successfully retained the antibacterial properties of MPE, as great inhibitory activity was observed against the Gram-negative bacterium *E. coli* and the Gram-positive bacterium *S. aureus*.

Despite the partial aggregation of the liposomes after freeze-drying, the drying process plays an important role in extending the long-term usability of the liposomes. SEM analysis confirmed the good stability of the freeze-dried liposomes, which otherwise exhibited a deformed spherical shape with no possible degradation. Through TGA/DSC and FTIR analyses, liposomes were found to increase the thermal stability of the encapsulated MPE and exhibit good chemical compatibility, as a negligible effect of chemical interactions between the encapsulated MPE and the lipids of the phospholipid bilayer of the liposomes was observed.

Therefore, MPE-encapsulated liposomes represent a stable, high-value-added formulation that enables the sustained release of bioactive substances from MPE with outstanding antibacterial properties for potential use in various applications. As MPE is a rich source of various bioactive substances, products in the cosmetics industry could be further enriched in this way, especially due to the content of ellagic acid, which is very important as a skin whitening agent and is also effective against wrinkles and aging. On the other hand, gallic acid is a powerful antioxidant and promotes the anti-inflammatory effect by reducing vasodilation and redness. Catechin also has antioxidant properties and inhibits hyperpigmentation and aging processes. In addition, liposomes are biocompatible and resemble a biological membrane. Thus, MPE-loaded liposomes represent a highly adaptable versatile platform to produce enriched cosmetic formulations (e.g., cosmetic creams and lotions), as they ensure the uniform release of bioactive substances from the liposomes and better penetration. Although the obtained results demonstrate the promising physicochemical properties of MPE-loaded liposomes and their potential for topical and related applications, the present study did not include biological safety or cytotoxicity assessments. Therefore, further investigations, including *in vitro* toxicity studies using relevant normal cell models, are necessary to confirm the safety and suitability of these formulations for cosmetic and pharmaceutical applications. Accordingly, the potential advantages of MPE-loaded liposomes should be regarded as promising but requiring additional experimental validation. In addition, the negative impact of mango peels on the environment could be reduced, which also has a significant impact on the circular economy. However, further studies are still required, particularly concerning long-term storage stability as a single product and as a component of various products such as cosmetic creams and lotions or food supplements. Further studies evaluating skin compatibility, skin penetration, and cosmetic efficacy are warranted to confirm their practical application.

## Figures and Tables

**Table 1:** Characteristics of liposomal formulations in terms of size, PDI, and zeta potential prepared by different synthesis methods.

Method	Organic Solvent	Mean Particle Size [nm]	PDI	Zeta Potential [mV]
Shaking with 3 mm glass beads	EtOH	453.97 ± 16.65	0.765 ± 0.106	−50.57 ± 2.42
Shaking with 5 mm glass beads		581.83 ± 44.77	0.499 ± 0.144	−50.63 ± 0.45
Sonication		591.67 ± 12.03	0.726 ± 0.388	−85.10 ± 1.06

**Table 2:** Characteristics of liposomal formulations in terms of size, PDI, and zeta potential after shaking with 5 mm glass beads for different time intervals.

Method	Organic Solvent	Shaking Time [h]	Mean Particle Size [nm]	PDI	Zeta Potential [mV]
Shaking with 5 mm glass beads	EtOH	2	581.83 ± 44.77	0.499 ± 0.144	−50.63 ± 0.45
		5	437.47 ± 2.76	0.298 ± 0.091	−50.23 ± 0.91
		24	142.53 ± 3.41	0.262 ± 0.015	−54.67 ± 4.11

**Table 3:** Characteristics of CIP- and MPE-loaded liposomes using different organic solvents.

Organic Solvent		Encapsulated Substance	Mean Particle Size [nm]	PDI	Zeta Potential [mV]
Polar solvent	EtOH	-	142.53 ± 3.41	0.262 ± 0.015	−54.67 ± 4.11
		CIP	147.20 ± 5.51	0.216 ± 0.007	−49.27 ± 3.66
		MPE	288.33 ± 3.96	0.290 ± 0.018	−55.17 ± 2.41
Non-polar solvent	TCM	-	137.40 ± 0.70	0.207 ± 0.010	−51.17 ± 1.82
		CIP	128.10 ± 1.95	0.162 ± 0.023	−53.63 ± 2.37
		MPE	382.23 ± 11.26	0.366 ± 0.043	−52.60 ± 3.70
Mixture	TCM:EtOH (1:1)	-	158.90 ± 1.87	0.369 ± 0.013	−56.67 ± 3.69
		CIP	176.20 ± 1.10	0.329 ± 0.020	−55.53 ± 5.18
		MPE	330.13 ± 10.06	0.401 ± 0.018	−53.83 ± 3.58

**Table 4:** Characteristics of liposomes with encapsulated MPE of different concentrations.

Concentration of MPE [mg/mL]	Size [nm]	PDI	Zeta Potential [mV]	Encapsulation Efficiency [%]	Loading Capacity [mg MPE/mg Dry Liposomes]
0.5	266.17 ± 7.63	0.464 ± 0.055	−51.23 ± 2.80	14.0 ± 2.0	0.008 ± 0.001
1.0	273.73 ± 7.51	0.367 ± 0.035	−55.83 ± 2.80	15.0 ± 3.5	0.018 ± 0.004
2.5	288.33 ± 3.96	0.290 ± 0.018	−55.17 ± 2.41	53.7 ± 2.7	0.161 ± 0.008
3.0	326.10 ± 2.35	0.311 ± 0.019	−53.50 ± 3.81	51.9 ± 1.9	0.187 ± 0.007
5.0	494.37 ± 5.86	0.408 ± 0.017	−50.37 ± 3.52	39.3 ± 1.4	0.235 ± 0.005
7.5	520.47 ± 43.46	0.401 ± 0.065	−49.17 ± 2.55	30.3 ± 2.4	0.279 ± 0.012

**Table 5:** Kinetic parameters of the *in vitro* release of encapsulated CIP and MPE from the liposomal formulations and the passage of the bioactive compounds in free form through the dialysis membrane fitted to four mathematical models (*k*_0_-zero-order release constant [%/min]; *k*_1_—first-order release constant [min^−1^]; *k*_H_—Higuchi model release constant [%/min^1/2^]; *k*_KP_—Korsmeyer–Peppas model release constant [min^−n^]; *n*—release exponent; *R*^2^—coefficient of determination).

Model	Zero-Order		First-Order		Higuchi		Korsmeyer–Peppas			Release Behavior
	*R* ^2^	*k*_0_	*R* ^2^	*k*_1_	*R* ^2^	*k*_H_	*R* ^2^	*k*_KP_	*n*	
Free CIP	0.207	0.019	0.994	0.013	-	-	0.740	28.427	0.177	Fickian diffusion mechanism
Encapsulated CIP	0.215	0.019	0.986	0.011	-	-	0.708	21.336	0.201	Fickian diffusion mechanism
Free MPE	0.475	0.027	0.984	0.006	0.463	2.739	0.892	12.577	0.274	Fickian diffusion mechanism
Encapsulated MPE	0.500	0.018	0.963	0.007	0.388	1.885	0.918	9.479	0.260	Fickian diffusion mechanism

**Table 6:** Bacterial reduction percentage of free MPE and CIP and MPE- and CIP-loaded liposomes.

Time [h]	Bacterial Reduction Percentage [%]							
	*E. coli*				*S. aureus*			
	MPE-Loaded Liposomes	Free MPE	CIP-Loaded Liposomes	Free CIP	MPE-Loaded Liposomes	Free MPE	CIP-Loaded Liposomes	Free CIP
3	89.68 ± 2.16	94.84 ± 3.62	99.97 ± 1.17	99.99 ± 1.01	87.97 ± 3.19	97.53 ± 2.74	99.94 ± 1.03	99.97 ± 0.94
6	97.18 ± 3.61	97.68 ± 2.14	100.00 ± 0.00	100.00 ± 0.00	97.00 ± 2.40	98.88 ± 2.36	100.00 ± 0.00	100.00 ± 0.00
24	99.46 ± 2.11	99.19 ± 2.02	100.00 ± 0.00	100.00 ± 0.00	98.89 ± 3.27	99.17 ± 3.02	100.00 ± 0.00	100.00 ± 0.00

## Data Availability

The data presented in this study are available on request from the corresponding author.

## Abbreviations

The following abbreviations are used in this manuscript:

CIP	Ciprofloxacin
DCM	Dichloromethane
DLS	Dynamic light scattering
DSC	Differential scanning calorimetry
DW	Dry weight
Et_2_O	Diethyl ether
EtOH	Ethanol
FTIR	Fourier transform infrared spectroscopy
GAE	Gallic acid equivalent
GRAS	Generally recognized as safe
GUV	Giant unilamellar vesicles
IPA	Isopropyl alcohol
LC-MS/MS	Liquid chromatography–mass spectrometry
LUV	Large unilamellar vesicles
MeOH	Methanol
MLVs	multilamellar vesicles
MPE	Mango (*Mangifera indica* L.) peel extract
PAC	Proanthocyanidins
PBS	Phosphate-buffered saline
PDI	Polydispersity index
SEM	Scanning electron microscopy
SUV	Small unilamellar vesicles
TCM	Chloroform (trichloromethane)
TGA	Thermogravimetric analysis
UAE	Ultrasound-assisted extraction
